# Analysis of the main active ingredients and bioactivities of essential oil from *Osmanthus fragrans* Var. *thunbergii* using a complex network approach

**DOI:** 10.1186/s12918-017-0523-0

**Published:** 2017-12-28

**Authors:** Le Wang, Nana Tan, Jiayao Hu, Huan Wang, Dongzhu Duan, Lin Ma, Jian Xiao, Xiaoling Wang

**Affiliations:** Shaanxi Key Laboratory of Phytochemistry, College of Chemistry and Chemical Engineering, Baoji University of Arts and Sciences, Baoji, Shaanxi 721013 China

**Keywords:** *Osmanthus fragrans* Var. *thunbergii*, Essential oil, Complex network, Active ingredients, Anti-tumor effect, Neuroprotective effect

## Abstract

**Background:**

*Osmanthus fragrans* has been used as folk medicine for thousands of years. The extracts of *Osmanthus fragrans* flowers were reported to have various bioactivities including free radical scavenging, anti-inflammation, neuroprotection and antitumor effects. However, there is still lack of knowledge about its essential oil.

**Methods:**

In this work, we analyzed the chemical composition of the essential oil from *Osmanthus fragrans* var. *thunbergii* by GC-MS. A complex network approach was applied to investigate the interrelationships between the ingredients, target proteins, and related pathways for the essential oil. Statistical characteristics of the networks were further studied to explore the main active ingredients and potential bioactivities of *O. fragrans* var. *thunbergii* essential oil.

**Results:**

A total of 44 ingredients were selected from the chemical composition of *O. fragrans* var. *thunbergii* essential oil, and that 191 potential target proteins together with 70 pathways were collected for these compounds. An ingredient-target-pathway network was constructed based on these data and showed scale-free property as well as power-law degree distribution. Eugenol and geraniol were screened as main active ingredients with much higher degree values. Potential neuroprotective and anti-tumor effect of the essential oil were also found. A core subnetwork was extracted from the ingredient-target-pathway network, and indicated that eugenol and geraniol contributed most to the neuroprotection of this essential oil. Furthermore, a pathway-based protein association network was built and exhibited small-world property. MAPK1 and MAPK3 were considered as key proteins with highest scores of centrality indices, which might play an important role in the anti-tumor effect of the essential oil.

**Conclusions:**

This work predicted the main active ingredients and bioactivities of *O. fragrans* var. *thunbergii* essential oil, which would benefit the development and utilization of *Osmanthus fragrans* flowers. The application of complex network theory was proved to be effective in bioactivities studies of essential oil. Moreover, it provides a novel strategy for exploring the molecular mechanisms of traditional medicines.

**Electronic supplementary material:**

The online version of this article (10.1186/s12918-017-0523-0) contains supplementary material, which is available to authorized users.

## Background

*Osmanthus fragrans*, also known as sweet olive or fragrant olive, is one of the most famous flowers in China due to attractive color and strong fragrance. Various varieties of *Osmanthus fragrans* flowers have been developed, and divided into four groups, Semperfloren, Thunbergii, Latifolius and Aurantiacus [1]. The cultivation and production of *O. fragrans* var. *thunbergii* group was more abundant than those of other groups [2]. This variation is considered to have higher market value and widely used in food chemistry [3].

*Osmanthus fragrans* flowers have been used as folk medicine for a long time in the treatment of rheumatism, cough and stomachache [4]. Extracts of *Osmanthus fragrans* flowers have been reported to exhibit various bioactivities both in vitro and in vivo. Water extract of the flower showed a potential to suppress TGF-β1-induced pulmonary fibrosis in human lung fibroblasts cells [5]. Acetonic extract of the flower exhibited antioxidant activity and melanogenesis inhibitory effect in murine melanoma cells [6]. Ethanol extract of the flower was reported to have neuroprotective and free radical scavenging effects [7]. Moreover, it also showed the ability of reducing allergic airway inflammation and oxidative stress in mice [8].

Essential oil is the volatile oil extracted from plants with strong aromatic components and distinctive odour, which has been widely used as a major raw material of perfume or cosmetic [9]. The essential oil is an excellent source of bioactive compounds, and exhibits various activities such as antioxidant and antimicrobial effects [10]. Therefore, it is considered to be partly responsible for the biomedical functions of plants [11]. Previous reports about *O. fragrans* var. *thunbergii* essential oil were mainly focused on its chemical composition. A broad group of low-molecular compounds were found in this essential oil, including linalool oxide, ocimene, 2,6-octadien-1-ol, 3,7-dimethyl-2-butanone, 3,7-dimethyl-*β*-ionone, and so on [12, 13]. However, the main active ingredients and bioactivities of this essential oil were still not fully elucidated.

One of the main causes for difficulties in the bioactivity study of *O. fragrans* var. *thunbergii* essential oil is the complexity of its chemical composition. Recently, complex network approach has emerged in the pharmacological researches, which was also known as “network pharmacology” or “system pharmacology” [14, 15]. This methodology holds a significant potential for extracting biological information from large amounts of chemical data [16], and enables to predict the target profiles and pharmacological actions of herbal compounds [17, 18]. For instance, Chandran et al. performed a network to evaluate the immunomodulatory activity of *Withania somnifera*, and revealed a series of novel bioactive immune target combinations. Chen et al. constructed a multi-parameter network model on the basis of three important parameters to tentatively explain the anti-fibrosis mechanism of herbal medicine *Sophora flavescens* [19]. Luo et al. used systems pharmacology strategies for anticancer drug discovery based on natural products [20]. In addition, Gogoi et al. developed a network pharmacology-based virtual screening of natural products from *Clerodendrum* species for identification of novel anti-cancer therapeutics [21]. These studies demonstrate that complex network approach has real potential for bioactivity study of natural products [22, 23].

In this study, we applied the complex network method to evaluate the main active ingredients and bioactivities of *O. fragrans* var. *thunbergii* essential oil. GC-MS was used to analyze ingredients of this essential oil. An ingredient-target-pathway network was constructed, and that statistical characteristics of the network were calculated to determine the key nodes. The nodes associated with the key pathway were extracted and reorganized as a core subnetwork to explore the underlying mechanism. Furthermore, a pathway-based protein association network was also built to investigate interrelationships between target proteins of *O. fragrans* var. *thunbergii* essential oil.

## Methods

### Plant material and sample preparation

Fresh flowers of *O. fragrans* var. *thunbergii* were collected from the campus of Baoji University of Art and Science (106°18′ E, 33°35’N, 618 m altitude), Baoji, China, in late September, 2015. The plant species was identified and confirmed by Prof. Xiaomei Wang, Shaanxi Key Laboratory of Phytochemistry, Baoji University of Art and Sciences. The flowers (BJWLXY-CC-SKLP150915) were dried in the shade at 25 °C until the humidity lower than 30%, and conserved in an airtight polyethylene bag at −20 °C. Samples (10 g) were extracted by steam distillation. The distilled liquid was then extracted with *n*-hexane, and dehydrated with anhydrous magnesium sulfate, followed by evaporation. Residual essential oil was stored at −20 °C until use.

### Chemical ingredients database building

GC-MS was used to analyze chemical ingredients of *O. fragrans* var. *thunbergii* essential oil. The essential oil was diluted in *n*-hexane (≥99%, GC grade, Sigma-Aldrich), and filtrated using a 0.22 μm filter. A blank (*n*-hexane) was prepared equally as the essential oil sample, and used as control sample. A measure of 1 μL sample was injected into a Trace 1300 GC coupled with an ISQ LT MS (Thermo, USA). Chromatographic separation was accomplished on a TG-5 column (30 m × 0.25 mm i.d.; film thickness: 0.25 μm; Thermo, USA). Temperature programming was set to 60 °C held for 2 min, then rose to 120 °C at 3 °C/min, maintained for 4 min, finally increased to 290 °C at 3 °C/min and held for 2 min. The inlet temperature was kept at 280 °C and ion source temperature was 270 °C. Helium was used as carrier gas at a constant flow rate of 1 mL/min. Electron impact ionization source (70 eV) was used at full scan mode (*m/z* 50-550).

Ingredients of *O. fragrans* var. *thunbergii* essential oil were identified by NIST 14 (National Institute of Standards and Technology, Gaithersburg, MD, USA) with a similarity of more than 70%. Retention indexes (RI) was also evaluated, using a saturated *n*-alkane mixture (C9-C36, Sigma Chemical, St. Louis, MO, USA). RI value for each component was calculated by AMDIS version 2.70 (Automated Mass Spectrometry Deconvolution and Identification System).

Lipinski’s five rules were applied to evaluate drug-like properties of the identified compounds in *O. fragrans* var. *thunbergii* essential oil [24]. A few important pharmaceutical properties were investigated using Pubchem (https://pubchem.ncbi.nlm.nih.gov/) and TCMSP (http://ibts.hkbu.edu.hk/LSP/tcmsp.php) [25], including molecular weight (MW < 500), number of donor atoms for H-bonds (HBD < =5), number of acceptor atoms for H-bonds (HBA < =10), and octanol-water partition coefficient (Mlog P < =5).

## Collection of target proteins and pathways analysis

Potential target proteins of the ingredients in *O. fragrans* var. *thunbergii* essential oil were collected using cyber tools such as SuperPred (http://prediction.charite.de/) and DrugBank (https://www.drugbank.ca/). Targets having interaction data for less than five compounds have been removed in the database. Information of all the selected proteins were converted and uniformed by Uniprot (http://www.uniprot.org/). In addition, ingredients without any target proteins were excluded.

Pathway analysis was performed on the selected target proteins utilizing KEGG pathway database (http://www.kegg.jp/kegg/). The database contains information about signal transduction, biological processes of cells, and homologous conservative path information of more than 700 species. The queried species in this study is *Homo sapiens*. Pathway analysis was carried out with a *P*-value threshold of 0.01.

## Network construction

The collected data were analyzed using complex network method. First, an ingredient-target-pathway network was constructed based on their interrelationships, designed to screen main active components and bioactivities of *O. fragrans* var. *thunbergii* essential oil. In this network, nodes represented ingredients, target proteins or pathways collected in previous steps, respectively. If a protein was the hit target of any ingredients, or involved in any pathways, connections were made between these nodes. Subsequently, targets and ingredients connected to the most important biological pathway were extracted from the ingredient-target-pathway network, and reconstructed as a core subnetwork to explore the underlying mechanism. Moreover, a pathway-based protein association network was built to evaluate the closeness of interaction between target proteins. If two proteins were both involved in common pathways, a connection was made between them. Isolated nodes were excluded from the network. All the networks for *O. fragrans* var. *thunbergii* essential oil were constructed and visualized by Pajek ver. 2.00 (Batagelj and Mrvar, 2009).

### Statistical analysis of the network

A series of parameters of the networks for *O. fragrans* var. *thunbergii* essential oil were investigated for more interpretation. Degree (*k*) was first calculated and ranked to evaluate the importance of a node. The degree *k*_*i*_ indicates the number of edges connecting to the node *i*. Mean value of *k*_*i*_ for all nodes in the network is defined as average degree, denoted as ⟨*k*⟩. Degree distribution, the proportion of randomly selected nodes with a certain number of connections, was represented as *P(k)*.

Average path length (L) refers to the mean distance between two nodes, which is averaged over all pairs of nodes. Diameter (D) is the maximum distance between any pair of nodes. Clustering coefficient (C) is the ratio of all existing edges between the neighbors and the maximum number of edges possible between these neighbors [26]. C is defined as following:

1 $$ {\mathrm{C}}_{\mathrm{i}}=\frac{2{\mathrm{e}}_{\mathrm{i}}}{{\mathrm{k}}_{\mathrm{i}}\left({\mathrm{k}}_{\mathrm{i}}-1\right)}\ \left({\mathrm{k}}_{\mathrm{i}}\ge 2\right) $$

where e_ij_ is the numbers of edges from node *i* to *j*.

Three centrality indices of nodes in the pathway-based protein association network were further evaluated, including degree centrality (*C*_*d*_), betweenness centrality (*C*_*b*_) and closeness centrality (*C*_*c*_). *C*_*d*_ is the proportion of other nodes adjacent to a node. *C*_*b*_ is the total number of shortest paths going through a node. *C*_*c*_ is the number of other nodes divided by the sum of the distances between a node and all the other nodes. The equations are listed as follows:

2 $$ {C}_d=\frac{{\mathrm{k}}_{\mathrm{i}}}{\mathrm{N}-1} $$

3 $$ {C}_b={\sum}_{\mathrm{j}\left(<k\right)}^{\mathrm{N}}{\sum}_{\mathrm{k}}^{\mathrm{N}}\frac{\mathrm{gjk}\left(\mathrm{i}\right)}{\mathrm{gjk}} $$

4 $$ {C}_c=\frac{\mathrm{N}-1}{\sum \limits_{\mathrm{j}=1}^{\mathrm{N}}{\mathrm{d}}_{\mathrm{ij}}} $$

where N is the total number of nodes in the network, g_jk_ is the numbers of geodesics connecting nodes *j* and *k*, and that d_ij_ is the shortest path length from node *i* to *j*. All the parameters were calculated and visualized using MATLAB 2009a (The MathWorks Inc., Natick, MA, USA).

## Results

### Chemical composition of *O. fragrans* Var. *thunbergii* essential oil

The yield of *O. fragrans* var. *thunbergii* essential oil was 0.19% (*w*/w, on a dry weight basis). Pharmaceutical potentials of herbal medicines are determined by their chemical compositions [27]. In the present study, a total of 91 compounds were identified from the essential oil, including alcohols, ketones, esters, hydrocarbons, terpenes, aldehydes, acids, and so on. Total ions chromatogram (TIC) of the sample was shown in Additional file 1, and that chemical information of identified compounds was listed in Additional file 2. Alcohols and ketones were major ingredients of the essential oil. This essential oil has highest concentration of dihydro-*β*-ionol, followed by *β*-ionone, (2,6,6-trimethyl-2-hydroxycyclohexylidene) acetic acid lactone and *γ*-decalactone. In order to narrow the scope of potential active ingredients, pharmaceutical properties of these compounds were researched. After the preliminary screening, 79 ingredients were reserved for the next step.

In previous reports, Xin et al. characterize the volatiles in flowers of four cultivar groups of sweet osmanthus (Wuhan, China), and that 71 compounds were found in Thunbergii cultivars. *β*-ionone, *cis*-linalool oxide (furan), trans-linalool oxide (furan) and linalool were the most common volatiles [3]. Hu et al. identified 52 components from *O. fragrans* var. *thunbergii* essential oil (Xianning, China), and that 1,2-epoxy linalool was the main component. Lei et al. identified 45 compounds in water-soluble essential oil components from *O. fragrans* var. *thunbergii* flowers (Hangzhou, China), which was rich in linalool oxide, 4-methoxyphenethyl and 4-hydroxyphenethyl alcohol [28]. Chemical composition of *O. fragrans* var. *thunbergii* essential oil revealed in the present study was generally consistent with other reports, but differed in percentages of individual components. This might because that compounds in *O. fragrans* var. *thunbergii* flowers varied among different geographical origins and flowering stages [2, 29].

### Target proteins and related pathways

Proteins are crucial for herbal medicines to achieve therapeutic effects in vivo. In order to explore potential bioactivities, we collected target proteins for the selected ingredients of *O. fragrans* var. *thunbergii* essential oil. A total of 191 target proteins were obtained (Additional file 3), including ornithine decarboxylases, adenosine receptors, hydroxyacid oxidases, and so on. It suggested that this essential oil might have various bioactivities. In addition, we excluded ingredients with no hit targets. Only 44 ingredients were reserved for further study (Additional file 3).

Pathway analysis was applied on target proteins of *O. fragrans* var. *thunbergii* essential oil for further interpretation, and the results showed that these proteins were involved in 70 biological pathways (Additional file 3), calcium signaling pathway, nitrogen metabolism, pentose and glucuronate interconversions, cytochrome P450, and so on. These pathways were critical in several important biological processes such as signal transduction, drug metabolism and energy metabolism, which indicated that *O. fragrans* var. *thunbergii* essential oil might have various bioactivities.

### The ingredient-target-pathway network

**Fig. 1:**
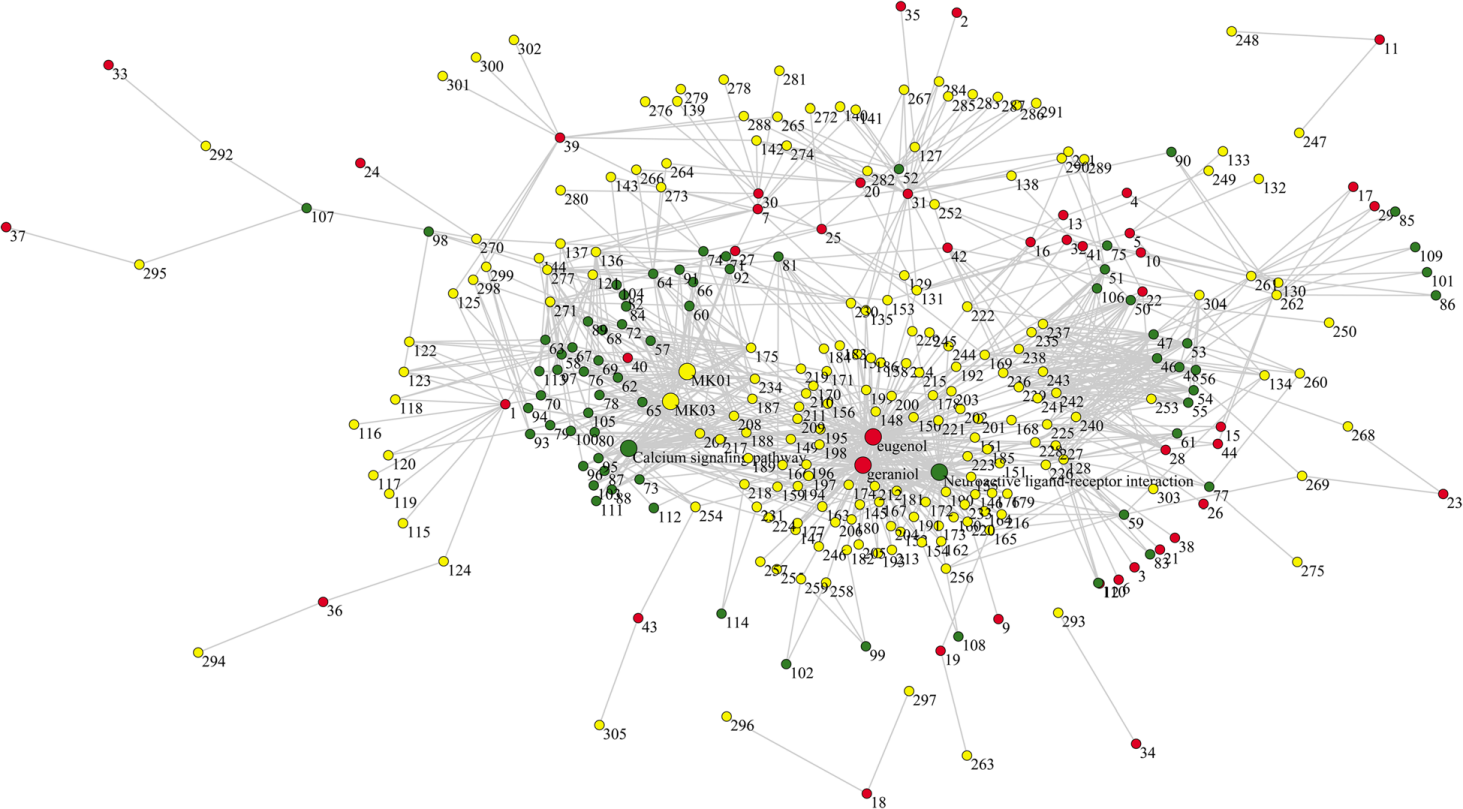
Ingredient-target-pathway network of *O. fragrans* var. *thunbergii* essential oil. The network consisted of 44 compounds (red nodes), 191 hit targets (yellow nodes), 70 related pathways (green nodes), and 879 connections. Numbers and names of nodes are listed in Additional file 3

**Fig. 2:**
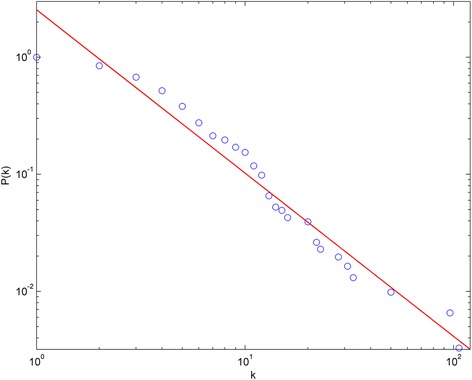
Degree distribution of the ingredient-target-pathway network. *k* represents degree values, and that *P(k)* indicates degree distribution

An ingredient-target-pathway network was built for *O. fragrans* var. *thunbergii* essential oil (Fig. 1), consisted of 305 nodes and 879 connections. The nodes included 44 ingredients, 191 target proteins, and 70 pathways. Degree distribution was first investigated as the basic property of a network. Degree values of all nodes were calculated and listed in Additional file 2. ⟨*k*⟩ of the network was 5.76. *P(k)* of the network was shown in Fig. 2. It is obvious that some nodes had much higher degree values, whereas others only had a few connections. In general, the network exhibited approximate scale-free property and power-law degree distribution, indicating that key nodes played a much more important role in the ingredient-target-pathway network of *O. fragrans* var. *thunbergii* essential oil.

### Main active ingredients of *O. fragrans* Var. *thunbergii* essential oil

As interaction between small molecule and protein plays a critical role in modulating the intrinsic biological processes, the information related to protein targets and small molecule has been highly valued by biomedical and pharmaceutical sciences. Target proteins are often those important ones in the development of specific diseases within the organism. Perturbing their functions by druggable molecules will help to cure the disease or relieve the symptoms [30]. Therefore, more attention was paid to highly connected ingredients of *O. fragrans* var. *thunbergii* essential oil in this study.

**Fig. 3:**
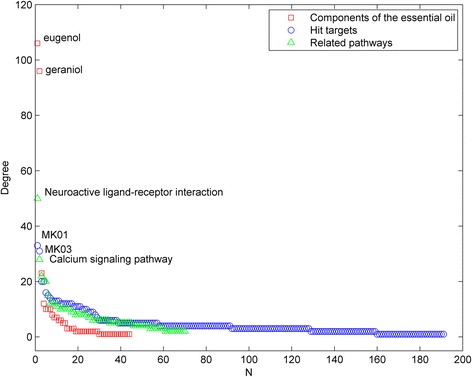
Degree values (*k*) of all nodes in the ingredient-target-pathway network, shown in descending order

In order to explore key nodes of the network, degree values of all nodes were ranked in a descending order (Fig. 3). Among all the 44 ingredients, eugenol (*k* = 106) and geraniol (*k* = 96) had most connections with target proteins, and were screened as key ingredients of *O. fragrans* var. *thunbergii* essential oil. Eugenol and geraniol were reported to exert multiple pharmacological actions. For instance, eugenol exhibited potential to inhibit ehrlich ascites and solid carcinoma [31]. Immunomodulatory and anti-inflammatory effects of eugenol were also found in clove extract [32]. Geraniol in essential oil of plants was reported to modulate DNA synthesis and potentiates 5-fluorouracil efficacy on human colon tumor xenografts [33]. Experimental evidences showed the therapeutic or preventive effects of geraniol on other different types of cancer, such as breast, lung, pancreatic, and hepatic cancer [34]. Geraniol could also inhibit prostate cancer growth by targeting cell cycle and apoptosis pathways [35]. In addition, geraniol in plant essential oil also exhibited anti-microbial activity against many microorganisms [36], which might be induced by causing leakage of K^+^ and Mg^2+^ ions from microorganism cells through changes in the compositions of the cell membranes [37].

### Potential anti-tumor effect of *O. fragrans* Var. *thunbergii* essential oil

In the target proteins of *O. fragrans* var. *thunbergii* essential oil, MAPK1 (MK01*, k* = 33) and MAPK3 (MK03*, k* = 31) had much higher degree values than others (Fig. 3). This indicated that the two proteins were associated with most ingredients and pathways in the network. They were considered as hub nodes and had significant impact on bioactivities of the essential oil. MAPK1 and MAPK3 were members of MAPKs family, which played an important role in MAPK/ERK cascade. Once universally activated, this pathway could block apoptosis, whereas the inhibition of MAPK/ERK is closely associated with anti-tumor effect [38]. MAPK1 and MAPK3 have been reported to be associated with various types of cancer, such as prostate cancer, colorectal cancer and gastric cancer [39–41].

A few compounds in this essential oil also showed a potential strong tumor-inhibiting activity through some other target proteins. p53 is one of the most intensively studied tumor suppressor proteins, with mutations that lead to loss of wild-type p53 activity frequently detected in many different tumor types [42]. In this study, p53 was the target protein of *cis*-anethol. It was involved in 15 pathways in the network, which were all closely correlated to tumor progression, such as apoptosis, pancreatic cancer, glioma, and melanoma. This protein, together with the hub proteins MAPK1 and MAPK3, all belong to MAPK signaling pathway (*k* = 12).

EGFR is another important protein in relation to tumor development [43]. Signaling by EGFR plays a key role in tumorigenesis prompted efforts to target this receptor in anticancer therapy [44]. It was the target protein of the selected main active ingredients, geraniol and eugenol. This protein was involved in 18 pathways in the network, most of which were tumor-related, such as ErbB signaling pathway, bladder cancer, endometrial cancer and colorectal cancer.

In addition, pathway analysis showed that several target proteins of *O. fragrans* var. *thunbergii* essential oil were involved in many tumor-related pathways (Additional file 2), including cytochrome P450 (*k* = 22), non-small cell lung cancer (*k* = 10), prostate cancer (*k* = 8), small cell lung cancer (*k* = 7), VEGF signaling pathway (*k* = 6), prostate thyroid cancer (*k* = 6), Wnt signaling pathway (*k* = 5), tyrosine metabolism (*k* = 4), and so on. These data suggested a potential anti-tumor effect of *O. fragrans* var. *thunbergii* essential oil.

There were also many reports about the anti-tumor effect of *Osmanthus fragrans*. Compounds isolated from ethyl acetate extract of *Osmanthus fragrans* var. *aurantiacus* flower could inhibit the growth of human colon cancer cell line HCT-116 [45]. Pomolic acid, isolated from flowers of *O. fragrans* var. *aurantiacus* Makino, could induce apoptosis in SK-OV-3 human ovarian adenocarcinoma cells [46]. These reports were consistent with the assumption that *O. fragrans* var. *thunbergii* essential oil had a potential anti-tumor effect.

### Potential neuroprotective effect of *O. fragrans* Var. *thunbergii* essential oil

The key pathway node of the network was neuroactive ligand-receptor interaction (Fig. 3). This pathway was connected with a total of 50 proteins, which meant that more than a quarter of target proteins of *O. fragrans* var. *thunbergii* essential oil were involved in it. Neuroactive ligand-receptor interaction contains a collection of receptors and ligands, and is closely related to neurological function and neurodegeneration diseases [47]. Coincidentally, some target proteins of this essential oil were proved to be enriched in Alzheimer’s disease (*k* = 8, Additional file 2), one of the best known neurodegeneration diseases. Besides, it has been reported that ethanol extract of dried *O. fragrans* var. *thunbergii* flowers had neuroprotective effect in Wistar rat [7]. Extract of this flower was also found to protect D-galactose induced aging in mouse model [48]. These data suggested a possible neuroprotection of *O. fragrans* var. *thunbergii* essential oil.

### The core subnetwork

**Fig. 4:**
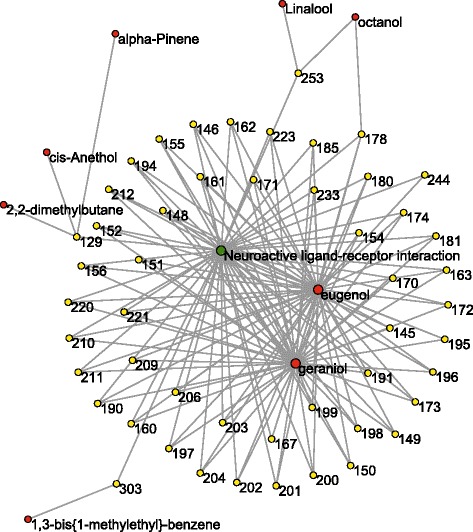
Core subnetwork of *O. fragrans* var. *thunbergii* essential oil. The network consisted of 59 nodes and 150 connections. Red nodes represent ingredients, yellow nodes indicate target proteins, and that green nodes refers to neuroactive ligand-receptor interaction. Numbers and names of the nodes are listed in Additional file 3

In order to get further understanding of the key pathway in the ingredient-target-pathway network, the nodes associated with neuroactive ligand-receptor interaction were extracted and reorganized as a core subnetwork (Fig. 4), which contained 8 ingredients, 50 proteins and 150 connections. Degree values of the network were also evaluated as mentioned above. Interestingly, eugenol (*k* = 47) and geraniol (*k* = 46) were also key nodes with much higher degree values, whereas ⟨*k*⟩ of the network was only 5.08. This result supported the importance of eugenol and geraniol for *O. fragrans* var. *thunbergii* essential oil.

Eugenol has been reported to protect neuronal cells from excitotoxic and oxidative injury [49]. Neuroprotective efficacy of eugenol was also found in acrylamide-induced neuropathy in rats [50]. Geraniol showed a neuroprotective effect in an acrylamide model of neurotoxicity in *Drosophila melanogaster* [51]. Additionally, geraniol was reported to be beneficial for the treatment of Parkinson’s disease associated with neuromuscular disability. These reports further confirmed that eugenol and geraniol played an important role in the neuroprotective effect of *O. fragrans* var. *thunbergii* essential oil.

### The pathway-based protein association network

**Fig. 5:**
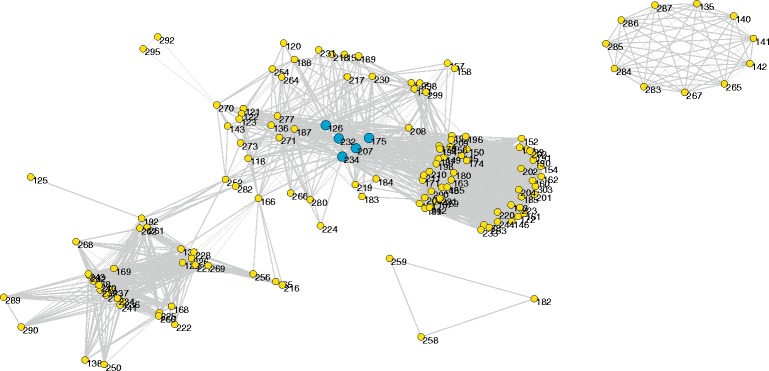
Pathway-based protein association network of *Osmanthus fragrans* var. *thunbergii* essential oil. The network consisted of 142 nodes and 2102 connections. Nodes represent target proteins of selected ingredients in *Osmanthus fragrans* var. *thunbergii* essential oil. Connections indicate that two proteins are involved in common pathway. Numbers and names of the nodes are listed in Additional file 3

Interrelationships between target proteins have great values in the network pharmacological study of traditional medicines [52]. In this section, a pathway-based protein association network was constructed for *O. fragrans* var. *thunbergii* essential oil to select key target proteins with strong interaction (Fig. 5). The network contained 142 nodes and 2102 connections. ⟨*k*⟩ of the network was 29.61. Degree distribution was evaluated and listed in Table 1. An uneven distribution was observed, as nodes with higher degree values accounted for a larger proportion, demonstrating that most target proteins of this essential oil were involved in common pathways.

**Table 1:** Statistics for the degree distribution of the pathway-based protein association network

*k*	Frequency ^a^	Frequency% ^b^	*k*	Frequency	Frequency%	*k*	Frequency	Frequency%
58	1	0.70	24	4	2.82	11	9	6.34
57	10	7.04	23	2	1.41	10	12	8.45
55	1	0.70	21	1	0.70	9	2	1.41
54	1	0.70	20	1	0.70	8	4	2.82
53	15	10.56	19	3	2.11	7	5	3.52
49	24	16.90	17	1	0.70	4	1	0.70
48	3	2.11	16	2	1.41	3	1	0.70
42	1	0.70	15	2	1.41	2	5	3.52
28	13	9.15	14	6	4.23	1	1	0.70
27	3	2.11	13	2	1.41			
26	3	2.11	12	3	2.11			

^a^Number of nodes with a specific degree value; ^b^ Percentage of nodes with a specific degree value in all the nodes

**Fig. 6:**
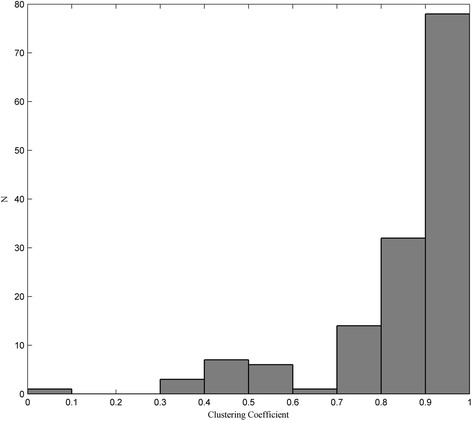
Distribution of clustering coefficient of the pathway-based protein association network. N represents number of nodes with a specific value of clustering coefficient

Clustering coefficient (C) represents cohesiveness of neighborhood for a node, ranging from 0 to 1 [26]. As Fig. 6 shown, most nodes of the pathway-based protein association network exhibited high values of C. On the other hand, L of the network was only 2.59. Short average path length and relatively high clustering coefficient suggested that the pathway-based protein association network had small-world property. This property demonstrated that the network was consisted of many small, but highly connected modules which combined in a hierarchical manner, and exhibited as larger, less cohesive units [53].

**Fig. 7:**
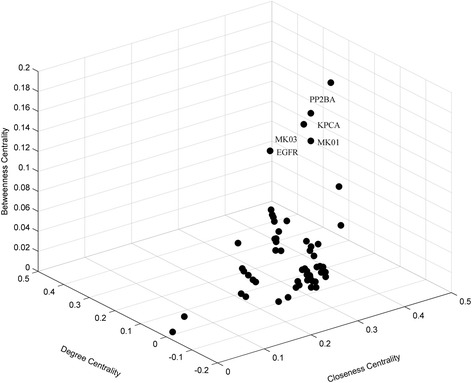
Three-dimensional diagram illustrating centrality indices of nodes in the pathway-based protein association network

In order to comprehensively evaluate the importance of individual components, three centrality indices, *C*_*d*_, *C*_*b*_ and *C*_*c*_ were calculated for nodes in the pathway-based protein association network. The top ten nodes with the highest scores of centrality indices were listed in Table 2. Subsequently, a three-dimensional diagram was drawn based on the centrality indices of all nodes (Fig. 7). Obviously, five nodes were outliers in the diagram, including KPCA, EGFR, PP2BA, MAPK1 and MAPK3. Centrality indices reflect the influence of a node on others through the network and how close a node is to others [54]. Therefore, these nodes were considered as key proteins in the pathway-based protein association network.

**Table 2:** Top ten nodes with the highest values of centrality indices in the pathway-based protein association network

No. ^a^	Protein name ^b^	C_d_^c^	No.	Protein name	C_b_^d^	No.	Protein name	C_c_^e^
175	EGFR	0.411	166	PGH2	0.177	207	KPCA	0.500
149	ADRB1	0.404	234	PP2BA	0.109	126	MK01	0.496
156	BKRB2	0.404	207	KPCA	0.093	232	MK03	0.496
170	DRD1	0.404	252	HCD2	0.082	234	PP2BA	0.496
194	ACM3	0.404	126	MK01	0.082	175	EGFR	0.447
195	ACM2	0.404	232	MK03	0.082	149	ADRB1	0.445
197	ACM5	0.404	175	EGFR	0.069	156	BKRB2	0.445
198	ACM1	0.404	282	TYRO	0.049	170	DRD1	0.445
209	5HT2C	0.404	192	AOFA	0.036	194	ACM3	0.445
210	5HT2A	0.404	261	ADH1B	0.027	195	ACM2	0.445

^a^Number of nodes are listed in Additional file 2; ^b^ Names of the target proteins are uniformed by Uniprot; ^c^ C_d_ represents values of degree centrality; ^d^ C_b_ represents values of betweenness centrality; ^e^ C_c_ represents values of closeness centrality

KPCA, also known as protein kinase C alpha type, is involved in several important processes such as cell proliferation, apoptosis, tumorigenesis, cardiac hypertrophy and angiogenesis. The expression levels of KPCA were reported to be closely related to esophageal carcinoma [39]. EGFR is the abbreviation of epidermal growth factor receptor. Mutation in EGFR could induce non-small cell lung cancer [41]. PP2BA (serine/threonine-protein phosphatase 2B catalytic subunit alpha isoform) has a role in the calmodulin activation of calcineurin, and is considered to be associated with human neurodegenerative disease [40]. These reports further supported the assumption that *O. fragrans* var. *thunbergii* essential oil had anti-tumor and neuroprotective effects.

Previous network analysis has revealed that MAPK1 (mitogen-activated protein kinase 1) and MAPK3 (mitogen-activated protein kinase 3) were hub nodes of the ingredient-target-pathway network. Furthermore, they were also screened as key proteins of the pathway-based protein association network. Consistency of the results indicated that the two proteins were not only associated with most ingredients and pathways, but also closely related to other target proteins of the essential oil. These data provided further support for the potential anti-tumor effect of *O. fragrans* var. *thunbergii* essential oil.

## Discussion

Although geraniol and eugenol together accounted for only 1.5% of the *O. fragrans* var. *thunbergii* essential oil (Additional file 2), these two compounds showed potential of strong bioactivity and acceptable cytotoxicity at the level of quantity according to previous reports. Geraniol originated from geranium essential oil was reported to show 312.5 μg/mL minimum inhibitory concentrations (MIC) against many species of bacteria such as *E. coli, Mycobacterium smegmatis, Pseudomonas aeruginosa, Salmonella typhi,* and *Yersinia enterocolitica*. Moreover, 100 μg/mL strong activity of geraniol was also found against *Staphylococcus epidermidis* and *Streptococcus mutans* [55]. Inhibitory concentration 50% values (IC_50_) for the anti-HSV effects of eugenol were 25.6 mg/mL and 16.2 mg/mL for HSV-1 and HSV-2 respectively, together with a maximum dose of 250 mg/mL for cytotoxicity test in the mouse model [56]. These reports supported the assumption that eugenol and geraniol were the main active ingredients of *O. fragrans* var. *thunbergii* essential oil.

## Conclusions

This study presents one of the first complex network analysis for evaluating active ingredients and bioactivities of plant essential oil. We analyzed the ingredients of *O. fragrans* var. *thunbergii* essential oil, and collected target proteins and related pathways for the selected compounds. Three networks were constructed based on these data to investigate the interrelationships. Topological Statistical analysis of these networks revealed that eugenol and geraniol were the main active ingredients of this essential oil. The essential oil also showed potential anti-tumor and neuroprotective effect, which were strongly supported by previous reports about *Osmanthus fragrans* [45, 46, 48, 49, 51]. Results of this work suggested a possible application of *O. fragrans* var. *thunbergii* essential oil in oncotherapy and neurological disorders, that would benefit the development and utilization of *Osmanthus fragrans* flowers. Complex network theory was also proved to be effective in bioactivities studies of essential oil. Moreover, it provides a novel strategy for exploring the molecular mechanisms of traditional medicines.

## Additional files

Additional file 1: Total ions chromatogram (TIC) of *O. fragrans* var. *thunbergii* essential oil (TIFF 325 kb)

Additional file 2: Chemical composition of *O. fragrans* var. *thunbergii* essential oil identified by GC-MS (DOCX 41 kb)

Additional file 3: Information of nodes in the ingredient-target-pathway network (DOCX 62 kb)
