# Supplementary figures and images for: Analysis of the main active ingredients and bioactivities of essential oil from Osmanthus fragrans Var. thunbergii using a complex network approach
Source: BMC Syst Biol. 2017 Dec 28;11:144. doi: 10.1186/s12918-017-0523-0 (PMC5745743; doi:10.1186/s12918-017-0523-0)

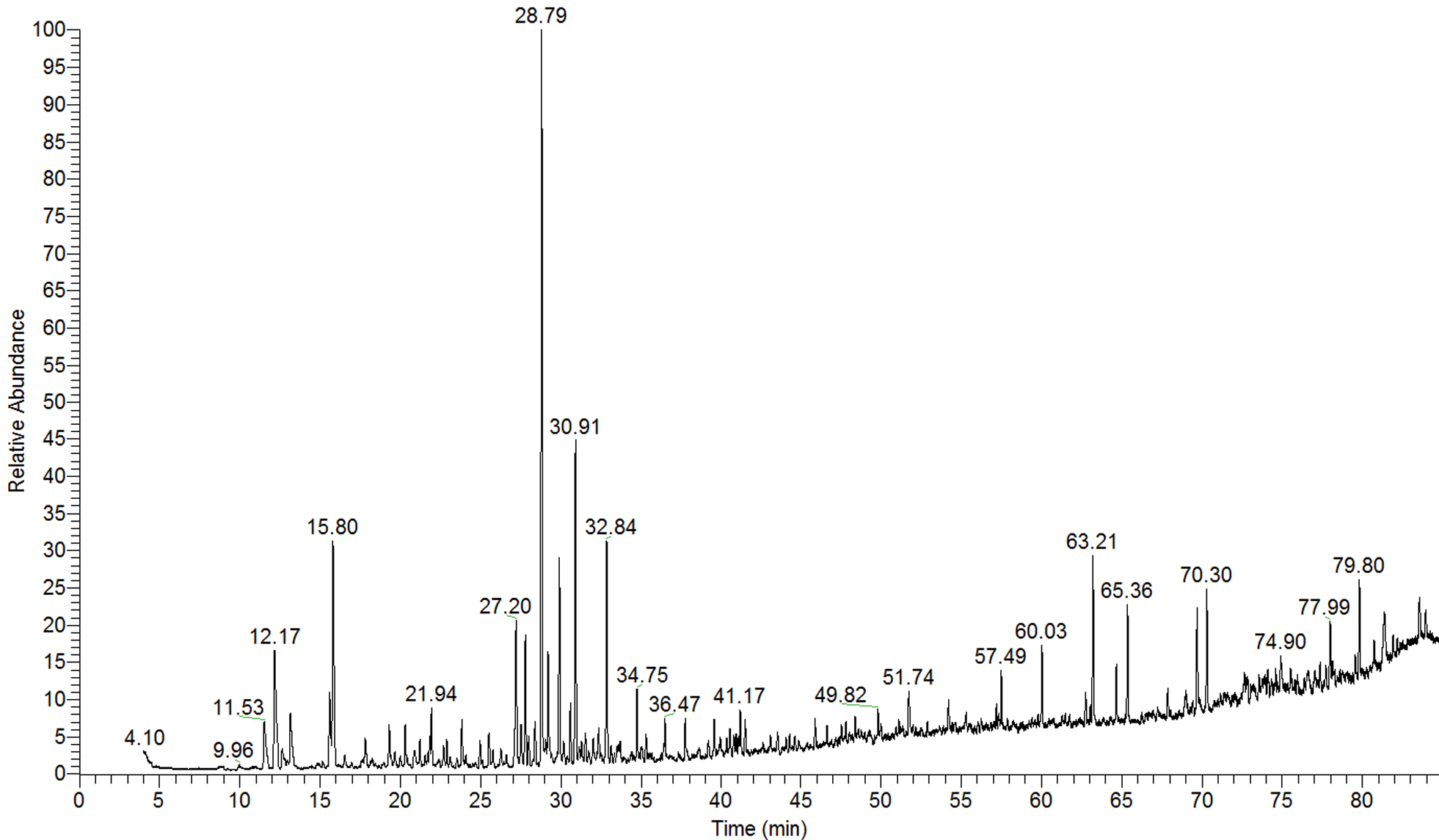

Supplement: Supplementary file 1 — Total ions chromatogram (TIC) of O. fragrans var. thunbergii essential oil (TIFF 325 kb) [file 12918_2017_523_MOESM1_ESM.tif]
