# Supplementary material for: Analysis of the main active ingredients and bioactivities of essential oil from Osmanthus fragrans Var. thunbergii using a complex network approach
Source: BMC Syst Biol. 2017 Dec 28;11:144. doi: 10.1186/s12918-017-0523-0 (PMC5745743; doi:10.1186/s12918-017-0523-0)
Supplement: Supplementary file 2 — Chemical composition of O. fragrans var. thunbergii essential oil identified by GC-MS (DOCX 41 kb) [file 12918_2017_523_MOESM2_ESM.docx]

**Additional file 2: Table S1** Chemical composition of *O. fragrans* var. *thunbergii* essential oil identified by GC-MS

| No.^a^ | Name ^b^ | *t*_R_  (min) | Molecular Formula | Molecular Weight | Composition ^c^  (%) | RI ^d^  (exp) | RI ^e^  (lit) |
| --- | --- | --- | --- | --- | --- | --- | --- |
| 1 | *γ*-Hexalactone | 4.22 | C_6_H_10_O_2_ | 114 | 0.0062 | 1023 | 1019 |
| 2 | 2-Propanone | 7.03 | C_3_H_6_O | 58 | 0.0005 | 478 | 475 |
| 3 | *cis*-5-Ethenyltetrahydro-α,α-5-trimethyl-2-furanmethanol | 12.15 | C_10_H_18_O_2_ | 170 | 2.7054 | 1092 | 1089 |
| 4 | Methyl ester acetic acid | 12.20 | C_3_H_6_O_2_ | 74 | 0.0005 | 503 | 506 |
| 5 | Linalool | 12.64 | C_10_H_18_O | 154 | 0.1261 | 1085 | 1082 |
| 6 | 2-Phenylethanol | 13.14 | C_8_H_10_O | 122 | 0.9306 | 1087 | 1084 |
| 7 | Ethyl methyl ketone | 13.53 | C_4_H_8_O | 72 | 0.0005 | 584 | 582 |
| 8 | Methyl methanoate | 14.58 | C_2_H_4_O_2_ | 60 | 0.0001 | 388 | 386 |
| 9 | Bicyclo[2.2.1]heptan-2-ol | 15.80 | C_7_H_12_O | 112 | 3.0082 | 934 | 930 |
| 10 | Terpinyl acetate | 16.51 | C_12_H_20_O_2_ | 196 | 0.1036 | 1325 | 1322 |
| 11 | *α*-Terpineol | 16.53 | C_10_H_18_O | 154 | 0.0833 | 1179 | 1172 |
| 12 | 2,3-Dihydrobenzofuran | 17.79 | C_8_H_8_O | 120 | 0.4659 | 1189 | 1188 |
| 13 | *β*-Cyclocitral | 17.79 | C_10_H_16_O | 152 | 0.3319 | 1198 | 1196 |
| 14 | 2-(1-Methylethenyl)-pyrazine | 17.79 | C_7_H_8_N_2_ | 120 | 0.4460 | 1085 | 1082 |
| 15 | 1-(2,5-Dimethyl-3-furanyl)-ethanone | 17.80 | C_8_H_10_O_2_ | 138 | 0.0193 | 1098 | 1095 |
| 16 | Geraniol | 19.30 | C_10_H_18_O | 154 | 0.7916 | 1235 | 1232 |
| 17 | *trans*-(+)-Isolimonene | 19.65 | C_10_H_16_ | 136 | 0.4090 | 949 | 947 |
| 18 | Tetrahydro-2-methyl-2-furanol | 20.45 | C_5_H_10_O_2_ | 102 | 0.0433 | 915 | 911 |
| 19 | 2-Methylnaphthalene | 20.88 | C_11_H_10_ | 142 | 0.0886 | 1299 | 1298 |
| 20 | Indole | 20.94 | C_8_H_7_N | 117 | 0.2057 | 1268 | 1265 |
| 21 | Acetic acid glacial | 21.56 | C_2_H_4_O_2_ | 60 | 0.0008 | 595 | 594 |
| 22 | *α*-Pinene | 21.70 | C_10_H_16_ | 136 | 0.3610 | 933 | 931 |
| 23 | 3,7,7-Trimethyl-bicyclo[4.1.0]hept-3-ene | 21.70 | C_10_H_16_ | 136 | 0.0438 | 1008 | 1005 |
| 24 | 2,2-Dimethyl-3-methylene-bicyclo[2.2.1]heptane | 21.71 | C_10_H_16_ | 136 | 0.2442 | 948 | 943 |
| 25 | 4'-Methoxyacetophenone | 21.82 | C_9_H_10_O_2_ | 150 | 0.3755 | 1338 | 1337 |
| 26 | Menthyl isovalerate | 21.93 | C_15_H_28_O_2_ | 240 | 1.4332 | 1539 | 1538 |
| 27 | 5-methyl-2-(1-methylethyl)cyclohexanol acetate | 21.94 | C_12_H_22_O_2_ | 198 | 1.0574 | 1298 | 1294 |
| 28 | 2-Methoxy-4-methylphenol | 21.94 | C_8_H_10_O_2_ | 138 | 1.7157 | 1165 | 1162 |
| 29 | 2-Methoxy-4-(1-propen-1-yl)-phenol | 23.83 | C_10_H_12_O_2_ | 164 | 0.7298 | 1413 | 1410 |
| 30 | Eugenol | 23.83 | C_10_H_12_O_2_ | 164 | 0.7085 | 1365 | 1363 |
| 31 | 4-Allyl-2-methoxyphenyl acetate | 23.84 | C_12_H_14_O_3_ | 206 | 0.1524 | 1486 | 1482 |
| 32 | 3,4-Methylenedioxyacetophenone | 23.87 | C_9_H_8_O_3_ | 164 | 0.2196 | 1435 | 1437 |
| 33 | Ethyl phenylacetate | 24.59 | C_10_H_12_O_2_ | 164 | 0.0012 | 1213 | 1210 |
| 34 | 4-(2,6,6-Trimethyl-2-cyclohexen-1-yl)-3-buten-2-ol | 24.97 | C_13_H_22_O | 194 | 0.2264 | 1378 | 1376 |
| 35 | *p*-Diethylbenzene | 27.09 | C_10_H_14_ | 134 | 0.0198 | 1048 | 1046 |
| 36 | 1-Isoproyl-3-tert-butylbenzene | 27.20 | C_13_H_20_ | 176 | 1.2642 | 1399 | 1397 |
| 37 | 4-(2,6,6-Trimethyl-1-cyclohexen-1-yl)-2-butanone | 27.23 | C_13_H_22_O | 194 | 3.4099 | 1431 | 1428 |
| 38 | 4,7,7-Trimethyl-bicyclo[4.1.0]hept-2-ene | 27.79 | C_10_H_16_ | 136 | 2.4338 | 1019 | 1017 |
| 39 | *α*-Terpinene | 27.79 | C_10_H_16_ | 136 | 2.9019 | 1005 | 1008 |
| 40 | Dihydro-β-linalool | 28.79 | C_13_H_24_O | 196 | 17.0187 | 1450 | 1449 |
| No.^a^ | Name | *t*_R_  (min) | Molecular Formula | Molecular Weight | Composition ^b^  (%) | RI ^c^  (exp) | RI ^d^  (lit) |
| 41 | Acetovanillone | 29.07 | C_9_H_10_O_3_ | 166 | 0.0840 | 1445 | 1447 |
| 42 | Geranylacetone | 29.20 | C_13_H_22_O | 194 | 2.2584 | 1428 | 1426 |
| 43 | 4-Dodecanolide | 29.87 | C_12_H_22_O_2_ | 198 | 0.9985 | 1629 | 1627 |
| 44 | *γ*-Decalactone | 29.90 | C_10_H_18_O_2_ | 170 | 4.7237 | 1418 | 1414 |
| 45 | 4-Heptanolide | 29.94 | C_7_H_12_O_2_ | 128 | 0.8229 | 1115 | 1113 |
| 46 | 7,8-Epoxy-α-ionone | 30.16 | C_13_H_20_O_2_ | 208 | 0.2853 | 1475 | 1473 |
| 47 | 2-(4-Methoxyphenyl)-4-methyl-1,3-dioxolane | 30.59 | C_11_H_14_O_3_ | 194 | 1.1179 | 1513 | 1512 |
| 48 | *β*-Ionone | 30.92 | C_13_H_20_O | 192 | 7.3171 | 1465 | 1463 |
| 49 | 4-(1,1-Dimethylethyl)phenol | 31.14 | C_10_H_14_O | 150 | 0.2284 | 1258 | 1256 |
| 50 | *n*-Nonane | 31.52 | C_9_H_20_ | 128 | 0.2045 | 903 | 900 |
| 51 | *n*-Octane | 31.53 | C_8_H_18_ | 114 | 0.3105 | 805 | 800 |
| 52 | 2-Methylenesuccinic anhydride | 32.59 | C_5_H_4_O_3_ | 112 | 0.0003 | 968 | 967 |
| 53 | (2,6,6-Trimethyl-2-hydroxycyclohexylidene)acetic acid lactone | 32.86 | C_11_H_16_O_2_ | 180 | 6.1274 | 1476 | 1471 |
| 54 | *p*-Tolualdehyde | 33.59 | C_8_H_8_O | 120 | 0.0333 | 1075 | 1073 |
| 55 | 5-Isopropyl-2-methylphenol | 34.72 | C_10_H_14_O | 150 | 1.6981 | 1277 | 1278 |
| 56 | 2-Pinen-4-one | 34.73 | C_10_H_14_O | 150 | 1.8182 | 1186 | 1183 |
| 57 | *p*-Isopropylbenzyl alcohol | 34.74 | C_10_H_14_O | 150 | 0.7080 | 1268 | 1266 |
| 58 | 2,5-Dimethyl-3-isopropylpyrazine | 34.75 | C_9_H_14_N_2_ | 150 | 1.9786 | 1125 | 1122 |
| 59 | 4-(1E)-1,3-Butadien-1-yl-3,5,5-trimethyl-2-cyclohexen-1-one | 35.31 | C_13_H_18_O | 190 | 0.2136 | 1475 | 1473 |
| 60 | 2,2-Dimethylbutane | 36.38 | C_6_H_14_ | 86 | 0.1610 | 533 | 529 |
| 61 | (R)-(+)-Pulegone | 37.75 | C_10_H_16_O | 152 | 0.2696 | 1216 | 1214 |
| 62 | *cis*-Anethol | 39.55 | C_10_H_12_O | 148 | 0.2207 | 1254 | 1251 |
| 63 | Methyl pyrrole-2-carboxylate | 40.16 | C_6_H_7_NO_2_ | 125 | 0.0008 | 2059 | 2058 |
| 64 | 2-Isopropylbenzaldehyde | 40.33 | C_10_H_12_O | 148 | 0.5834 | 1235 | 1230 |
| 65 | 3-Isopropylbenzaldehyde | 40.34 | C_10_H_12_O | 148 | 0.0754 | 1236 | 1234 |
| 66 | Resorcine | 40.37 | C_6_H_6_O_2_ | 110 | 0.0009 | 1369 | 1368 |
| 67 | Tetradecane | 40.55 | C_14_H_30_ | 198 | 0.9112 | 1405 | 1400 |
| 68 | Tridecanal | 41.06 | C_13_H_26_O | 198 | 0.4396 | 1488 | 1486 |
| 69 | 9-Hydroxy-5-megastigmen-4-one | 41.16 | C_13_H_22_O_2_ | 210 | 0.5794 | 1765 | 1762 |
| 70 | *m*-Di-iso-propylbenzene | 41.47 | C_12_H_18_ | 162 | 0.1768 | 1146 | 1142 |
| 71 | 1-(1,1-Dimethylethyl)-3-ethylbenzene | 41.47 | C_12_H_18_ | 162 | 0.2973 | 1138 | 1136 |
| 72 | 3-ethoxy-4-hydroxyallylbenzene | 43.06 | C_11_H_14_O_2_ | 178 | 0.4798 | 1495 | 1492 |
| 73 | Tetrahydrothiophen-3-one | 43.39 | C_4_H_6_OS | 102 | 0.0002 | 905 | 902 |
| 74 | Tridecane | 44.31 | C_13_H_28_ | 184 | 0.1461 | 1306 | 1300 |
| 75 | Undecane | 51.10 | C_11_H_24_ | 156 | 0.1550 | 1102 | 1100 |
| 76 | Tetradecanal | 51.72 | C_14_H_28_O | 212 | 1.5039 | 1595 | 1592 |
| 77 | Undecanal | 51.74 | C_11_H_22_O | 170 | 0.7295 | 1265 | 1261 |
| 78 | cyclohexanol | 51.76 | C_6_H_12_O | 100 | 0.1025 | 885 | 881 |
| 79 | 1-Tetradecanol | 57.49 | C_14_H_30_O | 214 | 0.4464 | 1677 | 1675 |
| 80 | 1-Undecanol | 57.49 | C_11_H_24_O | 172 | 1.1082 | 1355 | 1356 |
| 81 | Pentadecane | 60.02 | C_15_H_32_ | 212 | 1.4946 | 1504 | 1500 |
| 82 | Hexadecane | 60.04 | C_16_H_34_ | 226 | 2.0082 | 1603 | 1600 |
| No.^a^ | Name | *t*_R_  (min) | Molecular Formula | Molecular Weight | Composition ^b^  (%) | RI ^c^  (exp) | RI ^d^  (lit) |
| 83 | Dodecane | 62.75 | C_12_H_26_ | 170 | 0.2565 | 1202 | 1200 |
| 84 | Methacrolein | 64.68 | C_4_H_6_O | 70 | 0.0211 | 556 | 553 |
| 85 | 1-Pentadecanol | 64.68 | C_15_H_32_O | 228 | 2.5013 | 1795 | 1791 |
| 86 | Isobutyraldehyde | 64.76 | C_4_H_8_O | 72 | 0.0002 | 533 | 531 |
| 87 | Tetracosane | 65.37 | C_24_H_50_ | 338 | 3.1296 | 2403 | 2400 |
| 88 | 1-Tridecanol | 69.69 | C_13_H_28_O | 200 | 1.7483 | 1588 | 1586 |
| 89 | 1-Dodecanol | 69.70 | C_12_H_26_O | 186 | 1.9414 | 1458 | 1457 |
| 90 | Eicosane | 70.30 | C_20_H_42_ | 282 | 2.3476 | 2002 | 2000 |
| 91 | 6-Methyl-β-ionone | 79.80 | C_14_H_22_O | 206 | 0.9558 | 1585 | 1583 |

a, Numbers were sorted by retention time of each ingredient; b, Ingredients were identified by similarity of mass spectra and retention indices; c, Relative abundance of each ingredient; d, RI (retention indices) was measured relative to *n*-alkanes (C9~C36) on the non-polar TG-5 column under condition listed in the Methods section; e, RI originated from previous literatures.
