# Supplementary material for: Analysis of the main active ingredients and bioactivities of essential oil from Osmanthus fragrans Var. thunbergii using a complex network approach
Source: BMC Syst Biol. 2017 Dec 28;11:144. doi: 10.1186/s12918-017-0523-0 (PMC5745743; doi:10.1186/s12918-017-0523-0)
Supplement: Supplementary file 3 — Information of nodes in the ingredient-target-pathway network (DOCX 62 kb) [file 12918_2017_523_MOESM3_ESM.docx]

**Additional file 3: Table S2**. Information of nodes in the ingredient-target-pathway network

| **No.** | **Ingredient** ^a^ | ***k*** | **No.** | **Pathway** ^b^ | ***k*** | **No.** | **Target** ^c^ | ***k*** |
| --- | --- | --- | --- | --- | --- | --- | --- | --- |
| 1 | *β*-ionone | 12 | 45 | Neuroactive ligand-receptor interaction | 50 | 115 | RABP1 | 1 |
| 2 | Acetone | 1 | 46 | Drug metabolism - cytochrome P450 | 22 | 116 | RARG | 1 |
| 3 | *γ*-decalactone | 1 | 47 | Retinol metabolism | 20 | 117 | RET4 | 1 |
| 4 | 2,2-dimethylbutane | 2 | 48 | Metabolism of xenobiotics by cytochrome P450 | 20 | 118 | RARB | 3 |
| 5 | Phenylethanol | 5 | 49 | Calcium signaling pathway | 28 | 119 | RABP2 | 1 |
| 6 | 2,3-dihydrobenzofuran | 1 | 50 | Drug metabolism - other enzymes | 15 | 120 | RARA | 2 |
| 7 | Indole | 10 | 51 | Androgen and estrogen metabolism | 12 | 121 | RXRA | 7 |
| 8 | Eugenol | 106 | 52 | Nitrogen metabolism | 11 | 122 | RXRG | 6 |
| 9 | Isobutyraldehyde | 1 | 53 | Pentose and glucuronate interconversions | 10 | 123 | RXRB | 6 |
| 10 | Methyl(ethyl) ketone | 2 | 54 | Ascorbate and aldarate metabolism | 10 | 124 | HAOX1 | 2 |
| 11 | 3-methylideneoxolane-2,5-dione | 2 | 55 | Porphyrin and chlorophyll metabolism | 10 | 125 | DCOR | 3 |
| 12 | 5-propyloxolan-2-one | 1 | 56 | Starch and sucrose metabolism | 10 | 126 | MK01 | 33 |
| 13 | Methyl acetate | 1 | 57 | Gap junction | 12 | 127 | LMNA | 5 |
| 14 | Geraniol | 96 | 58 | Non-small cell lung cancer | 10 | 128 | CP1A2 | 15 |
| 15 | 2-Methylnaphthalene | 3 | 59 | Arachidonic acid metabolism | 9 | 129 | TSHR | 4 |
| 16 | Octanol | 6 | 60 | Bladder cancer | 8 | 130 | TYDP1 | 5 |
| 17 | Tridecanal | 2 | 61 | Linoleic acid metabolism | 7 | 131 | GEMI | 6 |
| 18 | 1-Undecanol | 2 | 62 | T cell receptor signaling pathway | 9 | 132 | Q7Z4Z5 | 1 |
| 19 | Phenol,2-methoxy-4-(2-propen-1-yl)-, 1-acetate | 2 | 63 | Thyroid cancer | 6 | 133 | NU1M | 1 |
| 20 | Acetovanillone | 7 | 64 | MAPK signaling pathway | 12 | 134 | CP2C8 | 6 |
| 21 | 4-Hexanolide | 1 | 65 | Regulation of actin cytoskeleton | 11 | 135 | CAH2 | 5 |
| 22 | Linalool | 5 | 66 | Prostate cancer | 8 | 136 | GSK3B | 12 |
| 23 | 4'-Methoxyacetophenone | 2 | 67 | Small cell lung cancer | 7 | 137 | ITAL | 4 |
| 24 | *p*-Diethylbenzene | 1 | 68 | Endometrial cancer | 6 | 138 | EST1 | 2 |
| 25 | *cis*-Anethol | 6 | 69 | Natural killer cell mediated cytotoxicity | 8 | 139 | ADK | 1 |
| 26 | Bicyclo[2.2.1]heptan-2-ol | 1 | 70 | PPAR signaling pathway | 6 | 140 | CAH1 | 3 |
| 27 | Alpha-Pinene | 3 | 71 | Epithelial cell signaling in Helicobacter pylori infection | 6 | 141 | CAH4 | 3 |
| 28 | 5-Isopropyl-2-methylphenol | 10 | 72 | Pancreatic cancer | 6 | 142 | CAH6 | 5 |
| 29 | Tetradecanal | 2 | 73 | VEGF signaling pathway | 6 | 143 | CDK5 | 3 |
| 30 | 3,4-Methylenedioxyacetophenone | 7 | 74 | Alzheimer's disease | 8 | 144 | ITB2 | 4 |
| 31 | Resorcine | 23 | 75 | Tyrosine metabolism | 4 | 145 | AA2AR | 4 |
| 32 | Cyclohexanol | 3 | 76 | ErbB signaling pathway | 6 | 146 | AA1R | 3 |
| 33 | Ethyl phenylacetate | 1 | 77 | Caffeine metabolism | 3 | 147 | AA3R | 2 |
| 34 | *p*-Tolualdehyde | 1 | 78 | Focal adhesion | 8 | 148 | ADA1D | 4 |
| 35 | *α*-Terpinene | 1 | 79 | Acute myeloid leukemia | 5 | 149 | ADRB1 | 5 |
| 36 | 2,6,6-Trimethyl-1-cyclohexene-1-carboxaldehyde | 2 | 80 | Glioma | 5 | 150 | ADRB3 | 4 |
| 37 | 2-Methoxy-4-methylphenol | 1 | 81 | Cytokine-cytokine receptor interaction | 8 | 151 | ADA2A | 3 |
| **No.** | **Ingredient** | ***k*** | **No.** | **Pathway** | ***k*** | **No.** | **Target** | ***k*** |
| 38 | 4-Dodecanolide | 1 | 82 | B cell receptor signaling pathway | 5 | 152 | ADA2B | 3 |
| 39 | Phenol, 2-methoxy-4-(1-propenyl)- | 10 | 83 | Monoterpenoid biosynthesis | 2 | 153 | ANDR | 4 |
| 40 | 3-Acetyl-2,5-dimethyl furan | 1 | 84 | Axon guidance | 6 | 154 | AGTR2 | 4 |
| 41 | Benzene, 1,3-bis(1-methylethyl)- | 2 | 85 | 1- and 2-Methylnaphthalene degradation | 2 | 155 | ADRB2 | 4 |
| 42 | Phenol, p-tert-butyl- | 8 | 86 | 3-Chloroacrylic acid degradation | 2 | 156 | BKRB2 | 5 |
| 43 | Pulegone | 2 | 87 | Adherens junction | 5 | 157 | CCR2 | 3 |
| 44 | Naphthalene, 2-methyl- | 3 | 88 | Fc epsilon RI signaling pathway | 5 | 158 | CCR4 | 3 |
|  |  |  | 89 | Colorectal cancer | 5 | 159 | CCR5 | 3 |
|  |  |  | 90 | Fatty acid metabolism | 3 | 160 | CALCR | 3 |
|  |  |  | 91 | Toll-like receptor signaling pathway | 5 | 161 | CNR1 | 3 |
|  |  |  | 92 | Melanogenesis | 5 | 162 | CATG | 4 |
|  |  |  | 93 | Pathogenic Escherichia coli infection - EHEC | 4 | 163 | CCKAR | 4 |
|  |  |  | 94 | Pathogenic Escherichia coli infection - EPEC | 4 | 164 | ACES | 2 |
|  |  |  | 95 | GnRH signaling pathway | 5 | 165 | PGH1 | 5 |
|  |  |  | 96 | Amyotrophic lateral sclerosis (ALS) | 4 | 166 | PGH2 | 5 |
|  |  |  | 97 | Leukocyte transendothelial migration | 5 | 167 | CLTR1 | 4 |
|  |  |  | 98 | Adipocytokine signaling pathway | 4 | 168 | CP2D6 | 3 |
|  |  |  | 99 | Biosynthesis of steroids | 3 | 169 | CP3A4 | 9 |
|  |  |  | 100 | Melanoma | 4 | 170 | DRD1 | 5 |
|  |  |  | 101 | Bile acid biosynthesis | 2 | 171 | DRD2 | 4 |
|  |  |  | 102 | Terpenoid biosynthesis | 2 | 172 | DRD4 | 3 |
|  |  |  | 103 | Long-term potentiation | 4 | 173 | DRD3 | 3 |
|  |  |  | 104 | Chronic myeloid leukemia | 4 | 174 | EDNRA | 4 |
|  |  |  | 105 | Wnt signaling pathway | 5 | 175 | EGFR | 20 |
|  |  |  | 106 | Tryptophan metabolism | 3 | 176 | ESR1 | 2 |
|  |  |  | 107 | Glycerolipid metabolism | 3 | 177 | ESR2 | 2 |
|  |  |  | 108 | Renin-angiotensin system | 2 | 178 | GCR | 6 |
|  |  |  | 109 | Glycolysis / Gluconeogenesis | 2 | 179 | KCNH2 | 2 |
|  |  |  | 110 | Limonene and pinene degradation | 2 | 180 | HRH1 | 4 |
|  |  |  | 111 | Long-term depression | 3 | 181 | HRH2 | 4 |
|  |  |  | 112 | Urea cycle and metabolism of amino groups | 2 | 182 | HMDH | 3 |
|  |  |  | 113 | Apoptosis | 3 | 183 | CXCR2 | 4 |
|  |  |  | 114 | Primary immunodeficiency | 2 | 184 | CXCR1 | 4 |
|  |  |  |  |  |  | 185 | OPRK | 3 |
|  |  |  |  |  |  | 186 | ELNE | 2 |
|  |  |  |  |  |  | 187 | MK14 | 11 |
|  |  |  |  |  |  | 188 | MMP1 | 4 |
|  |  |  |  |  |  | 189 | MMP9 | 4 |
|  |  |  |  |  |  | 190 | MC3R | 3 |
|  |  |  |  |  |  | 191 | MC4R | 3 |
|  |  |  |  |  |  | 192 | AOFA | 6 |
|  |  |  |  |  |  | 193 | SC6A3 | 2 |
|  |  |  |  |  |  | 194 | ACM3 | 5 |
| **No.** | **Ingredient** | ***k*** | **No.** | **Pathway** | ***k*** | **No.** | **Target** | ***k*** |
|  |  |  |  |  |  | 195 | ACM2 | 5 |
|  |  |  |  |  |  | 196 | ACM4 | 4 |
|  |  |  |  |  |  | 197 | ACM5 | 5 |
|  |  |  |  |  |  | 198 | ACM1 | 5 |
|  |  |  |  |  |  | 199 | NK1R | 4 |
|  |  |  |  |  |  | 200 | NK2R | 4 |
|  |  |  |  |  |  | 201 | NPY1R | 3 |
|  |  |  |  |  |  | 202 | NPY2R | 3 |
|  |  |  |  |  |  | 203 | OPRD | 3 |
|  |  |  |  |  |  | 204 | OPRM | 3 |
|  |  |  |  |  |  | 205 | PDE5A | 2 |
|  |  |  |  |  |  | 206 | PTAFR | 4 |
|  |  |  |  |  |  | 207 | KPCA | 20 |
|  |  |  |  |  |  | 208 | ERBB2 | 11 |
|  |  |  |  |  |  | 209 | 5HT2C | 5 |
|  |  |  |  |  |  | 210 | 5HT2A | 5 |
|  |  |  |  |  |  | 211 | 5HT2B | 5 |
|  |  |  |  |  |  | 212 | 5HT6R | 4 |
|  |  |  |  |  |  | 213 | SC6A2 | 2 |
|  |  |  |  |  |  | 214 | SC6A4 | 2 |
|  |  |  |  |  |  | 215 | SGMR1 | 2 |
|  |  |  |  |  |  | 216 | THAS | 3 |
|  |  |  |  |  |  | 217 | FYN | 10 |
|  |  |  |  |  |  | 218 | LCK | 5 |
|  |  |  |  |  |  | 219 | VGFR1 | 4 |
|  |  |  |  |  |  | 220 | VIPR1 | 3 |
|  |  |  |  |  |  | 221 | V1AR | 4 |
|  |  |  |  |  |  | 222 | AL1A1 | 6 |
|  |  |  |  |  |  | 223 | ADA2C | 3 |
|  |  |  |  |  |  | 224 | CASP1 | 3 |
|  |  |  |  |  |  | 225 | CP2A6 | 8 |
|  |  |  |  |  |  | 226 | CP2CJ | 9 |
|  |  |  |  |  |  | 227 | CP2C9 | 9 |
|  |  |  |  |  |  | 228 | CP2E1 | 6 |
|  |  |  |  |  |  | 229 | ABC3G | 1 |
|  |  |  |  |  |  | 230 | IL8 | 6 |
|  |  |  |  |  |  | 231 | PTPRC | 4 |
|  |  |  |  |  |  | 232 | MK03 | 31 |
|  |  |  |  |  |  | 233 | MC5R | 3 |
|  |  |  |  |  |  | 234 | PP2BA | 14 |
|  |  |  |  |  |  | 235 | UD11 | 13 |
|  |  |  |  |  |  | 236 | UD110 | 12 |
|  |  |  |  |  |  | 237 | UD16 | 12 |
| **No.** | **Ingredient** | ***k*** | **No.** | **Pathway** | ***k*** | **No.** | **Target** | ***k*** |
|  |  |  |  |  |  | 238 | UD18 | 12 |
|  |  |  |  |  |  | 239 | UD19 | 12 |
|  |  |  |  |  |  | 240 | UD14 | 13 |
|  |  |  |  |  |  | 241 | UD2A1 | 13 |
|  |  |  |  |  |  | 242 | UDB15 | 11 |
|  |  |  |  |  |  | 243 | UDB17 | 10 |
|  |  |  |  |  |  | 244 | GBRB2 | 2 |
|  |  |  |  |  |  | 245 | TRPV2 | 1 |
|  |  |  |  |  |  | 246 | TRPV4 | 1 |
|  |  |  |  |  |  | 247 | FNTA | 1 |
|  |  |  |  |  |  | 248 | FNTB | 1 |
|  |  |  |  |  |  | 249 | DYN1 | 1 |
|  |  |  |  |  |  | 250 | EST2 | 1 |
|  |  |  |  |  |  | 251 | CBX1 | 3 |
|  |  |  |  |  |  | 252 | HCD2 | 5 |
|  |  |  |  |  |  | 253 | THB | 3 |
|  |  |  |  |  |  | 254 | PPARD | 5 |
|  |  |  |  |  |  | 255 | VDR | 1 |
|  |  |  |  |  |  | 256 | LOX15 | 3 |
|  |  |  |  |  |  | 257 | KMT2A | 1 |
|  |  |  |  |  |  | 258 | GGPPS | 3 |
|  |  |  |  |  |  | 259 | FDFT | 3 |
|  |  |  |  |  |  | 260 | CP2AD | 4 |
|  |  |  |  |  |  | 261 | ADH1B | 12 |
|  |  |  |  |  |  | 262 | ADH1A | 11 |
|  |  |  |  |  |  | 263 | ACRO | 1 |
|  |  |  |  |  |  | 264 | PTN7 | 2 |
|  |  |  |  |  |  | 265 | CAH5A | 4 |
|  |  |  |  |  |  | 266 | PTN5 | 2 |
|  |  |  |  |  |  | 267 | CAH7 | 3 |
|  |  |  |  |  |  | 268 | AK1C2 | 2 |
|  |  |  |  |  |  | 269 | AK1C3 | 3 |
|  |  |  |  |  |  | 270 | LIPL | 4 |
|  |  |  |  |  |  | 271 | P53 | 16 |
|  |  |  |  |  |  | 272 | AHR | 1 |
|  |  |  |  |  |  | 273 | TAU | 4 |
|  |  |  |  |  |  | 274 | RORG | 2 |
|  |  |  |  |  |  | 275 | TRPA1 | 1 |
|  |  |  |  |  |  | 276 | SENP7 | 1 |
|  |  |  |  |  |  | 277 | TF65 | 13 |
|  |  |  |  |  |  | 278 | SENP6 | 1 |
|  |  |  |  |  |  | 279 | NR0B1 | 1 |
|  |  |  |  |  |  | 280 | NFKB2 | 2 |
| **No.** | **Ingredient** | ***k*** | **No.** | **Pathway** | ***k*** | **No.** | **Target** | ***k*** |
|  |  |  |  |  |  | 281 | SENP8 | 1 |
|  |  |  |  |  |  | 282 | TYRO | 3 |
|  |  |  |  |  |  | 283 | CAH3 | 2 |
|  |  |  |  |  |  | 284 | CAH5B | 2 |
|  |  |  |  |  |  | 285 | CAH12 | 2 |
|  |  |  |  |  |  | 286 | CAH14 | 2 |
|  |  |  |  |  |  | 287 | CAH9 | 2 |
|  |  |  |  |  |  | 288 | NF2L2 | 2 |
|  |  |  |  |  |  | 289 | DHB1 | 2 |
|  |  |  |  |  |  | 290 | DHB2 | 2 |
|  |  |  |  |  |  | 291 | GABT | 1 |
|  |  |  |  |  |  | 292 | MGLL | 2 |
|  |  |  |  |  |  | 293 | B2L11 | 1 |
|  |  |  |  |  |  | 294 | OPSR | 1 |
|  |  |  |  |  |  | 295 | LIPP | 2 |
|  |  |  |  |  |  | 296 | F1D8Q5 | 1 |
|  |  |  |  |  |  | 297 | A0A024R5S8 | 1 |
|  |  |  |  |  |  | 298 | TBB2B | 4 |
|  |  |  |  |  |  | 299 | TBB1 | 4 |
|  |  |  |  |  |  | 300 | LGUL | 1 |
|  |  |  |  |  |  | 301 | BRSK1 | 1 |
|  |  |  |  |  |  | 302 | SGK2 | 1 |
|  |  |  |  |  |  | 303 | GBRA1 | 2 |
|  |  |  |  |  |  | 304 | UD17 | 10 |
|  |  |  |  |  |  | 305 | NR1H4 | 1 |

a, Ingredients of the essential oil from *O. fragrans* var. *Thunbergii*; b, Names of the pathways are uniformed by KEGG; c, Names of the target proteins are uniformed by Uniprot.
